# KIR-HLA class I combinations in JIA-associated uveitis

**DOI:** 10.1186/1546-0096-10-S1-A114

**Published:** 2012-07-13

**Authors:** Miriam Parsa, Deborah K McCurdy, Jennifer MP Woo, Angela Presson, Raj Rajalingam, Ralph Levinson, Gary Holland

**Affiliations:** 1 Mattel Children's UCLA, Los Angeles, CA, USA; 2 UCLA, Los Angeles, CA, USA

## Purpose

HLA class I-specific killer cell immunoglobulin-like receptors (KIR) gene and HLA gene combinations have been associated with autoimmune uveitis (e.g., birdshot chorioretinopathy (BCR) and Vogt-Koyanagi-Harada syndrome (VKH)). In oligoarticular juvenile idiopathic arthritis (JIA), uveitis is one of the most serious complications, affecting approximately 20% of patients. Multiple studies suggest there are HLA Class II associations with chronic anterior uveitis in JIA; however, there is a paucity of evidence demonstrating Class I associations. Given the importance of HLA Class I and KIR gene combinations in other forms of autoimmune uveitis, we hypothesized that an interplay between these genes may contribute to the pathogenesis of uveitis in JIA. To test this hypothesis, we are: 1) determining HLA Class I and KIR gene associations in children with JIA with and without uveitis and comparing them with healthy controls, and 2) determining associations between clinical course and HLA Class I - KIR gene associations.

## Methods

Study population included pediatric patients with JIA, with and without uveitis. DNA samples are typed for 16 KIR genes using a gene-specific PCR typing system. HLA-A, -B and -C typing are performed by sequence-specific oligonucleotide hybridization methods. We analyzed the presence or absence of 16 KIR genes in patients with uveitis, patients without uveitis, and published healthy controls (n = 429). The JIA clinical phenotype, including medication use and assessment of functional outcome by the Children’s Health Assessment Questionnaire (CHAQ), were compared with genotype. Data were analyzed by student’s t-test, linear regression, and multivariate logistic regression modeling. Further testable genetic hypotheses were generated with Weighted Gene Co-expression Network Analysis (WGCNA) or cluster analysis modeling.

## Results

A total of 29 (52% with uveitis) blood samples have been collected, and the 16 KIR genes have been genotyped. In previous studies, KIR3DL3, -3DP1, -2DL4, -3DL2 were considered to be framework genes and were present in all adults with autoimmune uveitis. These findings were replicated in our pediatric sample (p = 0.0001); there were 2 additional KIR genes present in 100% of the tested individuals (KIR2DL1, KIR2DP1). There was no association of KIR genotype in oligoarticular JIA patients with or without uveitis as compared with healthy control population nor an association of KIR genotype in oligoarticular JIA patients with uveitis compared to oligoarticular JIA patients without uveitis. Similarly, there was no association seen with KIR genotype and polyarticular JIA patients (n = 6) with or without uveitis versus healthy control population.

## Conclusion

In this small sample size, we did not find an association between KIR gene frequencies and JIA-associated uveitis. To reduce the possibility of a Type I error, we are expanding our sample size and we will compare the KIR genotypes to HLA types. Uveitis remains a significant risk for disability in JIA (e.g., cataracts, visual loss); therefore, developing a risk prediction model may allow for aggressive screening and disability prevention.

## Disclosure

Miriam Parsa: None; Deborah K. McCurdy: None; Jennifer M.P. Woo: None; Angela Presson: None; Raj Rajalingam: None; Ralph Levinson: None; Gary Holland: None.

